# Isometric Posterior Chain Peak Force Recovery Response Following Match-Play in Elite Youth Soccer Players: Associations with Relative Posterior Chain Strength

**DOI:** 10.3390/sports7100218

**Published:** 2019-10-01

**Authors:** Emma Constantine, Matt Taberner, Chris Richter, Matthew Willett, Daniel D. Cohen

**Affiliations:** 1 School of Sport, Exercise and Rehabilitation Sciences, University of Birmingham, Birmingham B15 2TT, UK; m.j.willett.1@bham.ac.uk; 2 Department of Sport Science and Medicine, Manchester City Women’s Football Club, Manchester M11 4TQ, UK; 3 Performance Department, Everton Football Club, Liverpool L26 3UE, UK; matthewtaberner@btinternet.com; 4 School of Sports and Exercise Sciences, Liverpool John Moore’s University, Liverpool L26 3UE, UK; 5 Sports Surgery Clinic, Dublin D09 C523, Ireland; mr.chris.richter@gmail.com; 6 Centre of Precision Rehabilitation for Spinal Pain (CPR Spine), School of Sport, Exercise and Rehabilitation Sciences, University of Birmingham, Birmingham B15 2TT, UK; 7 Faculty of Health Sciences, University of Santander (UDES), Bucaramanga 680001-680011, Colombia; danielcohen1971@gmail.com

**Keywords:** soccer, hamstring, recovery, isometric, monitoring

## Abstract

The purpose of this study was to determine changes in two tests of lower limb isometric posterior chain force (IPC-F) following 90 min of match-play in elite youth soccer players and the interaction between relative strength and recovery profile. 14 players (age: 16 ± 2 years) performed 3 × 3 second IPC-F tests unilaterally at 30° and 90° of knee and hip flexion pre- and post-match, +24 h, +48 h, and +72 h post-match. Peak force was recorded for both limbs, combined and expressed relative to bodyweight (N/kg). A two-way repeated measures analysis of variance was performed to determine differences in force output between joint angles, time intervals and subjects. As there was no interaction between angle and time (*p* = 0.260), we report the change between timepoints as mean ∆ in 90° + 30° IPC-F. Relative to pre-match IPC-F, there were significant decreases post (∆ = −18%; *p* > 0.001) and at +24 h (∆ = −8%; *p* = 0.040), no significant difference at +48 h (∆ = 0%; *p* = 0.992) and a significant increase at +72 h (∆ = +12%; *p* = 0.005). There was a large inter-individual variability in recovery profile at both angles and substantial differences between post-match deficits at 90° (−10.8%) compared to 30° (−20.7%). Higher pre-match IPC-F was correlated with the magnitude of IPC-F deficits at both angles and all time points (r = 0.56 to 0.70, *p* = < 0.01) except for post-match 90°. Regular IPC-F monitoring to determine the magnitude of match-induced fatigue and track recovery may help inform decision-making regarding modifications to individual players training load, particularly as there is a large inter-individual variability in response to competition. Further research is warranted to better understand and address the finding that stronger players showed larger force deficits and slower recovery following match-play.

## 1. Introduction

Hamstring strain injuries (HSIs) are amongst the most common injuries in professional soccer, accounting for approximately 12% of all muscular injuries recorded [1]. As inadequate hamstring strength or high levels of interlimb strength asymmetry [2,3] have been identified as modifiable risk factors, preseason strength screening is often implemented in soccer and other repeated sprint sports to identify players with deficits. This approach, however, fails to capture potential interactions between a player’s response to the demands of individual match-play or accumulated matches and HSI risk. Congested competition schedules are associated with a higher incidence of muscular injury to the hamstring complex [4,5]. This has prompted practitioners and researchers to assess the associations between training and competition load, recovery time, and injury risk [6,7,8,9]. During soccer match-play, repeated explosive movements are performed including high-speed running (HSR), sprinting, changes of direction, and acceleration and deceleration efforts [10], placing large metabolic and mechanical demands upon the hamstring muscle group. High-force eccentric actions have been shown to result in high levels of muscle damage [6,11], contributing to reductions in lower body neuromuscular function [12] during and up to 72 h following a single competitive soccer match [6,13,14,15]. It has been demonstrated that isometric peak force loss following eccentric exercise is directly related to the degree of muscle damage [16]. Therefore, in-season weekly or bi-weekly assessment of isometric hamstring strength represents a potential means for practitioners to define players “load response”—encompassing the magnitude of fatigue induced by match-play and the rate of recovery in the post-match period. By identifying abnormal fatigue or recovery responses, this information could help to inform decision-making around player training load management between matches. The assessment of isometric strength using a force platform, fixed dynamometer or pressure cuff is a practical and acceptable means to assess neuromuscular function at the posterior chain in the post-match period and is used as a weekly monitoring tool in a number of professional soccer teams.

McCall et al. [15] demonstrated, in professional adult soccer players, the reliability of a simple isometric posterior chain force (IPC-F) test performed on a force platform at 90° and 30° of hip and knee flexion and noted significant decrements in IPC-F immediately following match-play, also indicating both positions sensitivity to acute competition-induced fatigue. While the fatigue-recovery response of the IPC-F test at both 90° and 30° have not been examined in elite youth players, Wollin and colleagues evaluated IPC-F post-match and at 24 h, 48 h and 72 h using a fixed dynamometer test, and also reported significant deficits post-match and at 24 h with recovery at 48 h following a competitive match [17].

Intuitively, the magnitude of match-play posterior chain fatigue, characterised by IPC-F decrements, would be related to the volume of running load and specific actions within soccer match-play, yet to our knowledge only Nedelec et al. [11] has examined potential correlations or determinants of the magnitude of IPC-F decline post-match. They reported associations between number of tackles and IPC-F changes but no other associations between playing actions or components of match load. This contrasts with evidence from studies using the countermovement jump (CMJ) as a measure of neuromuscular fatigue, showing associations between specific components of player load and reductions in aspects of CMJ performance [11,18]. Importantly, Johnston et al. [19] observed that despite greater external match loads, elite youth rugby league players with greater high-intensity running ability and lower body strength (defined by 3RM back squat) experienced a smaller decrement in CMJ peak power in the post-match period. This suggests that physical qualities, including maximal muscle strength may mediate the magnitude of fatigue and rate of recovery in response to match-play. To our knowledge, correlations between posterior chain strength and the magnitude of acute and residual match-induced fatigue have not been examined. As fatigue is considered to be a risk factor for injury, contributing to higher incidence in the latter part of matches [11], understanding whether higher isometric posterior chain strength is protective against match-induced strength decline or not, is relevant to training prescription aimed at HSI risk reduction.

Therefore, the aims of this study were 1) to evaluate in elite youth soccer players, the fatigue- recovery profile of IPC-F at 30° and 90°, immediately post-match and daily in the subsequent 72 h and 2) to assess potential associations between pre-match IPC-F and the magnitude of match-induced decrements in IPC-F at these time points.

## 2. Methods

### 2.1. Subjects

14 outfield players (Age 16.9 ± 0.7 years; Height 178.3 ± 6.8 cm; Weight 73.2 ± 12.0 kg; Bodyfat 10.3 ± 1.4 %) were recruited from a professional English League One youth team. Testing was conducted between March 2017 and November 2017, spanning two consecutive seasons. Ethics approval was granted by both the University of Birmingham (No. CM23/02/17-1) and by Oldham Athletic Football Club Ltd (Oldham, UK).

Players participated if they (1) had no current or previous lower limb injury two months prior to testing [15,20], (2) had no pain at the time of testing, and (3) were not taking medications/drugs.

In addition, to improve the standardisation of the testing conditions, players tests were excluded from the analysis if (1) they had participated in a training session rated above 4 on the modified rating of perceived exertion scale (Foster, 1998) three days prior to testing [11,15], (2) reported elevated lower limb muscle soreness before testing (defined as the player scoring above 2 on the 1–7 “Likert scale of muscle soreness”) (Hooper et al. 1995), or (3) had not completed 90 minutes match-play.

Similar match exposure criteria have previously been used to assess the reliability and sensitivity of IPC-F testing in professional soccer players, with intraclass correlations above 0.86 reported on both dominant and non-dominant limbs [15].

### 2.2. Procedures

Players performed IPC-F tests unilaterally in both limbs at 30° and 90° of hip and knee flexion, on a single (vertical) axis force platform at a sampling frequency of 1000 Hz, (PS-2141, Pasco, Roseville, CA) pre-, post-, and at +24 h, +48 h and +72 h post match-play. Players did not participate in training and refrained from participating in vigorous activity for up to +72 h post-match. One week prior to testing, players performed two test familiarisation sessions, with verbal instructions piloted during this period. As a maximum of four substitutions per game are allowed at a professional league one youth level, seven outfield players were randomly selected from the starting 10 for pre-game assessments, ensuring that at least three of the players tested would complete 90 minutes of match-play. Consequently, data was obtained over five separate matches, and for any one player only one cycle of testing was included in the analysis. All matches were played on natural turf with dimensions of 101 × 68 m.

### 2.3. Testing Procedures

Prior to testing, players performed a standardised low intensity cycle ergometer (Keiser, Tetbury, UK) warm-up consisting of 5 minutes at resistance level 7 and 5 minutes at resistance level 10 at 90 revolutions per minute prior to testing at each time point except for post-match. IPC-F testing was performed within 5 minutes of the cessation of the warm-up at pre, +24 h, +48 h and +72 h, and between 5 and 15 minutes after the end of the game in the post-match test session.

**Figure 1:**
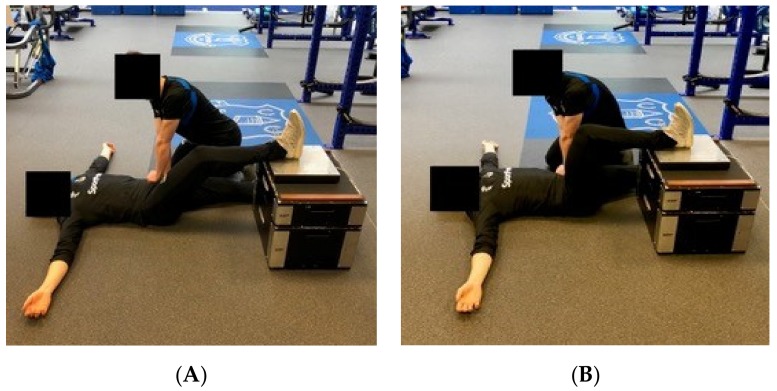
Isometric Posterior Chain Force (IPC-F) tests at 30° (**A**) and 90° (**B**).

The 30° and 90° IPC-F tests (Figure 1) were performed as described by McCall et al. [15], with the player lying supine on a mat, the heel of the testing limb placed on a force plate resting on a firm plinth with the testing angle set using a goniometer (Physio Parts, Twickenham, UK) and the non-testing limb relaxed and fully extended. The player was instructed to push the heel of the testing limb into the force plate exerting as much force as fast as possible whilst keeping the buttocks, hips, hands and head on the mat. External pressure was applied to the non-tested limb to limit extension. Standardised instructions and verbal encouragement were given during the procedure, and a verbal command of “3, 2, 1 GO” countdown given before the initiation of a maximal contraction which was held for 3 seconds, as per “3, 2, 1 relax”. The 30° test was performed first across all timepoints, players first performing three trials on the left limb, followed by three trials on the right. A rest of 10 seconds was allowed between trials on the same limb. This process was then repeated for the 90° tests. 60 seconds was allowed between the last test on a limb at 30° and the 1st test of the same limb at 90°. All testing was supervised by the same assessor—an experienced physiotherapist. IPC-F (Newtons; N) was calculated by summing the mean value of 3 trials on the right limb and the mean value of 3 trials on the left limb. This value was then divided by body mass to calculate relative IPC-F (Newtons per kilogram; N/kg). IPC-F output was adjusted to body mass in order to normalise the data set, highlighted as the most common way to account for the direct relationship between body size and strength [21], hence shank length was not taken into account in the present study. Body mass (kg) was assessed regularly as required by the elite player performance plan (EPPP) guidelines for growth and maturation screening [22].

### 2.4. Statistical Analysis

Data was analyzed using statistical parametric mapping (spm1d version 0.4) and tested for normal distribution (K2 test statistic) and subsequently examined for differences using a two-way repeated analysis of variance (ANOVA; as per spm1d.stats.anova2rm). If a significant difference was found for an effect (Angle, Time, Angle × Time), a post-hoc test was performed using a paired t-test without Bonferroni correction [23]. To examine the relationship between peak strength and recovery profile, the Pearson coefficient was calculated to determine the correlation between pre-match IPC-F and the change in IPC-F from pre to post-match, +24 h, +48 h and +72 h. To examine the variability within a testing session the coefficient of variation was also computed at every time point. Data are presented as mean ± standard deviation (SD). Significance level was set at *p* = 0.05. All data illustration and analysis was performed in MATLAB 2014a. To illustrate the data, a combination of a boxplot and violin plot (representation of the data’s distribution [kernel probability density]).

## 3. Results

14 outfield players fulfilled the inclusion criteria and completed the IPC-F testing at both 30° and 90° positions at all time points and were included in the analysis. The average coefficient of variation within the sessions was 0.075 (standard deviation = 0.084; 25th percentile = 0.032; 75th percentile = 0.090).

### 3.1. IPC-F at 30° and 90°

Table 1 reports the mean (SD) 30° and 90° total peak force (mean peak force across trials) relative to body weight (N/kg) at each time point. No differences in limb (*p* = 0.632) and no interactions were observed during analysis (limb-angle *p* = 0.484; limb-time *p* = 0.198; angle-time *p* = 0.275; limb-angle-time; *p* = 0.528). As such we report only findings of the summed peak force values of the left and right limb values—see Table 1 for the mean (SD) 30° and 90° total peak force (mean peak force across trials) relative to body weight (N/kg) at each time point. Pre-match relative peak force was significantly greater (*p* = 0.010) at 90° (6.5 ± 1.4 N/kg) than at 30° (5.8 ± 1.5 N/kg).

**Figure 2:**
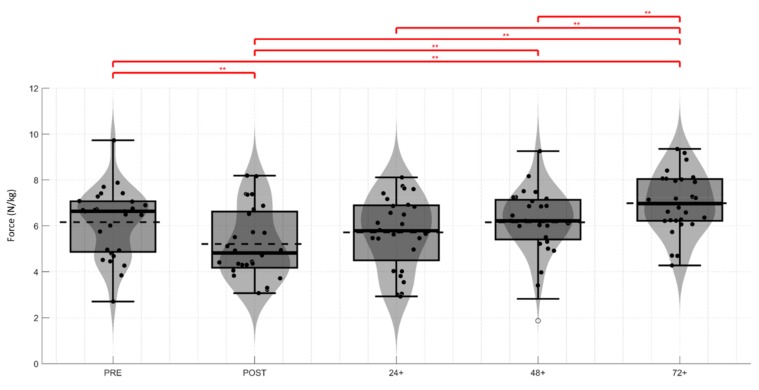
Mean change in relative IPC-F (N/kg) between each time point tested (Pre-match, Post-match, +24 h, +48 h and +72 h). Violin and box plot represent variation across the data sets for each player in relation to mean and median values. ** indicates significant difference: Pre to Post, Post to +72 h, +24 h to +72 h; *p =* 0.000, Pre to +72 h and post to +48 h; *p =* 0.004, +48 h to +72 h; *p =* 0.005.

As there was no interaction between angle and time (*p* = 0.275). Therefore, in further analysis of the change (Δ %) across timepoints, we evaluated mean of peak force at 90° + 30° (IPC-F). There were significant decreases in IPC-F between pre-match and: post (Δ = −18%; *p* = 0.001), +24 h (Δ = −8%; *p* = 0.04), +48 h (Δ = 0%; *p* = 0.992) and at +72 h (Δ = +12%; *p* = 0.005). Figure 2 shows the analysis of IPC-F across all time points.

### 3.2. Relationship between Pre-Match IPC-F and IPC-F Change Following Match-Play

**Figure 3:**
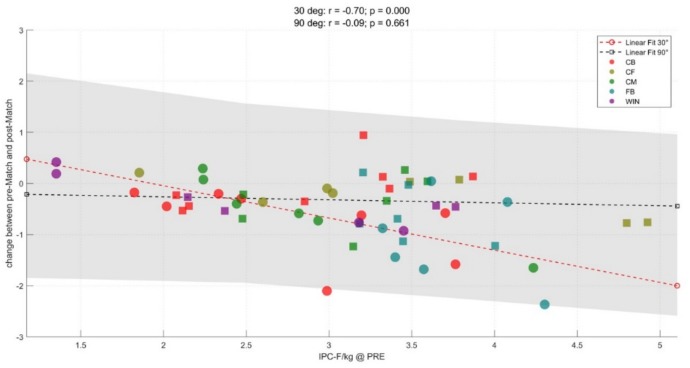
Correlation between pre-match IPC-F (N/kg) at 30° and 90° and Δ IPC-F post-match. CB = centre-back, CF = centre-forward, CM = centre-midfielder, FB = full-back, WIN = winger.

**Figure 4:**
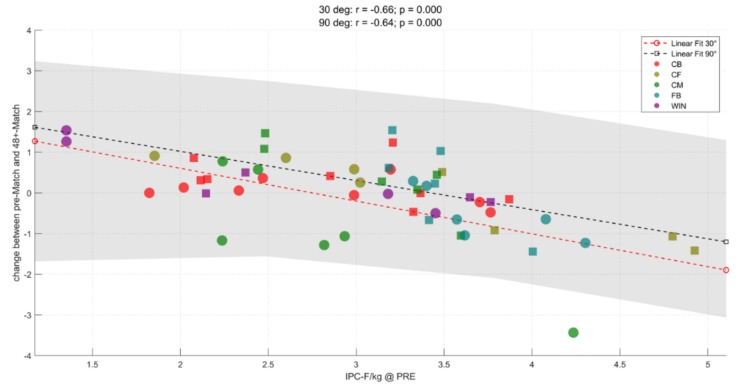
Correlation between pre-match IPC-F (N/kg) at 30° and 90° and Δ IPC-F at 48 h. CB = centre-back, CF = centre-forward, CM = centre-midfielder, FB = full-back, WIN = winger.

**Figure 5:**
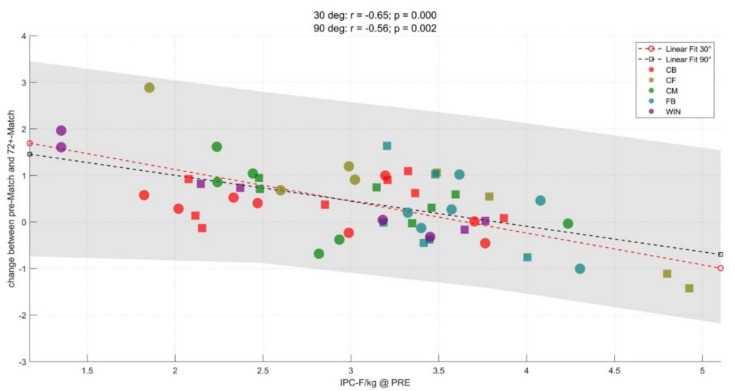
Correlation between pre-match IPC-F (N/kg) at 30° and 90° and Δ IPC-F at 72 h. CB = centre-back, CF = centre-forward, CM = centre-midfielder, FB = full-back, WIN = winger.

Figure 3, Figure 4 and Figure 5 illustrate the correlations between pre-match IPC-F in the 90° and 30° and change (Δ) in IPC-F in both tests at each time point assessed. Higher pre-match IPC-F 30° was positively correlated with the magnitude of IPC-F decline at all timepoints (r = −0.66 to 0.70; *p* < 0.001). Higher pre-match IPC-F 90° was positively correlated with the magnitude of IPC-F decline at 48 h and 72 h (r = 0.56–0.64; *p* < 0.01) but not post-match (r = 0.09, *p* > 0.050).

## 4. Discussion

We evaluated the effects of competitive match play on IPC-F assessed at two joint positions, immediately post-match and at +24 h, +48 h and +72 h later, in elite youth soccer players. We found significant reductions at both positions immediately post-match, at +24 h, recovery at +48 h, while at +72 h values were significantly higher than baseline. We also assessed the association between player’s relative posterior chain strength and the magnitude of force decrement observed at each time point and found that players with higher levels of pre-match posterior chain strength had larger reductions/slower recovery of IPC-F.

### 4.1. IPC-F Fatigue-Recovery Response and Angle-Specific Differences

Deficits in the immediate post-match period are characterised as acute fatigue and are principally related to ionic and metabolic disturbances [24]. In agreement with previous literature, evaluating the acute effects of match or simulated match-play on IPC-F in elite youth [17] and senior soccer players [15,25], we found a significant reduction in IPC-F post-match. The present study is the first to assess the IPC-F fatigue-recovery profile in elite youth players using the 90° and 30° tests, but the magnitude of decline post-match is similar to the 17.7% reported by Wollin et al. [17] in elite youth players evaluated using a fixed dynamometer. We evaluated both angles proposed by Schache et al. [26] and McCall et al. [15] as they differ in recruitment pattern, with a higher relative bicep femoris (BF) activation at 30° than 90° [27]. In the present study, there were notable differences in the IPC-F fatigue-recovery profile; particularly the substantially larger acute IPC-F decrement at 30° than 90° (20.7% vs 10.8%) and at 24 h (12.1% vs 3.1%), and the larger increment at +72 h (17.2% vs 9.2%) that potentially warrant further consideration. Previous research comparing the response to competitive match-play of both IPC-F at 30° and 90° in senior players found a larger decline in IPC-F at 30° than at 90° +24 h post-match (effect size at 30°; 0.91–1.08 at 90°; 0.67–0.77; [11]) or a similar magnitude of acute IPC-F decline [15]. Following a simulated match protocol, Matinlauri et al. [25], observed that semi-professional adult players had a larger decrement in IPC-F assessed in a 90° hip, 20° knee flexion test compared to that observed at 90°. The authors suggested that this was due to the 90°:20° position inducing a higher relative contribution of the BF and a greater knee extension—aligning with previous work showing greater acute fatigue in dynamic knee flexion at greater extension following simulated soccer competition [28]. The substantially larger response to acute competition induced fatigue in the 30° in the present study may therefore reflect the higher BF activation at that joint angle [27,29] in combination with greater relative fatigue in BF than in other posterior chain muscles active in the IPC tests. Interestingly, in the present study and that of Nedelec et al. [11], the timepoint at which the magnitude of 30° and 90° IPC-F changes (relative to pre-match baseline) were least divergent was at +48 h or “match-day +2” - the time-point at which IPC assessments are typically performed (at least in English Soccer). Deficits at this time point are related to the degree of mechanical damage and the proportion of fibres affected [16] and driven by exposure to the intense eccentric contractions [11,30] implicit in decelerations and change of directions. Overall, these data suggest that the tests have a similar capacity to detect residual deficits, but that the 30° or other tests, which put a greater demand on the BF, have marginally greater sensitivity. However, in deciding which test is “better” to implement, in the time pressured setting typical of weekly monitoring, other practical considerations come into play, such as the ease of setting up the 90° test. Nonetheless, if time/environment permits evaluating in both positions and assessing changes at the two angles within individuals could potentially provide information around the relative fatigue response across the posterior chain muscles differentially involved in these tests.

We observed a broadly similar pattern of IPC-F fatigue recovery profile in the +24 h to +72 h post-match period as previously reported, but with some aspects worthy of note. Firstly, while the acute (post-match) IPC-F deficit we observed at 90° was slightly lower than that previously reported in senior players at 90°, at +48 h we found no mean deficit and most players IPC-F returned to pre-match values, while players in those studies still showed significant deficits at +48 h (of~8%–10%) [11,24]. Our findings align more closely to that of Wollin et al. [17], who reported non-significant (~3%) deficits at +48 h in elite youth players of a similar mean age as players in the present study but used a fixed dynamometer. Intuitively, recovery from intense exercise is slower in older individuals, but contrasting our data and that of Wollin et al. [17] with previous findings in adults [11,25] raises the possibility that even in their early 20s, players may already experience a slower rate of recovery and larger/more persistent neuromuscular deficits following match-play, a possibility which warrants further investigation.

### 4.2. 72 h Post-Match—“Positive Adaptation”

A notable finding of the present study was that at 72 h, mean IPC-F was significantly higher than pre-match values, in contrast to a return to pre-match values [25] or persistent deficits [11] previously reported at this timepoint. It is important to note that unique to the present study, players were only included in the analysis if they did not perform any training or match-play in the +72 h post-match period. While this may be considered an artificial situation in elite football, it did allow us to describe the “pure” fatigue-recovery response of IPC-F in response to competitive match i.e. without the potentially confounding influence of additional training delaying recovery. Our data suggests that when combined with adequate recovery, match-play may provide a stimulus for posterior chain muscle strength development, measurable with these isometric tests. However, we acknowledge that this positive adaptation may have been inflated by factors such as; increased practice and familiarisation with tests, pre-match “baseline” values that did not represent full recovery from previous days training or priming/potentiation of IPC-F by match-play [31]. Nonetheless, while a novel finding with respect to IPC-F, the observation of a positive adaptation to competition does concur with Morgans et al. [32] who reported increased CMJ height and peak power three days post-competition in elite professional soccer players. They suggested that match-play and in particular HSR volume, with which positive adaptation was most strongly associated, represented the highest physiological load a player is exposed to, and is an important stimulus for muscle power adaptations. These findings however, might lead one to ask; if match-play represents a strong stimulus for neuromuscular adaptations, is the player adequately conditioned for it? Future research in this area is warranted.

### 4.3. Variation in IPC-F and VariaVariability in IPC-F Response

While our sample was too small to allow a robust statistical comparison of positional differences in IPC-F, there appeared to be positional trends in IPC-F; for example a full-back within the sample; Pre-match IPC-F at 30° = 6.7 N/kg and at 90° = 6.6 N/kg, compared with a central midfielder; at 30° = 4.7 N/kg and at 90° = 5.0 N/kg. These differences in IPC-F force could relate to the specific adaptations of imposed demands of training and match-play [33], influenced by positional differences in external load [34]. In elite soccer academies players develop a playing position from a young age based on technical, tactical and physical capabilities, which are further developed during their academy years [35]. For example, a full-backs match demands involve larger volumes of high-speed, sprint and high-acceleration distances [36] and would favour greater stimulus for adaptations in type 2 fibres and for force and power development. In contrast, central midfielders who cover greater total distance comprising of less high-speed and sprint distance [37], match stimulus would favour adaptations in type I fibres and improvements in oxidative capacity [38]. As in previous studies, we noted a large inter-individual variability in acute and residual IPC-F force deficits following real and simulated match-play [15,17]. The relationship between IPC-F deficits and external load have not been described, but it is likely that the large inter-individual variability in running loads in matches [5] and in particular, the volume of HSR is one of the determinants of the variability in the IPC-F fatigue-recovery profile. Our study was limited by the lack of micro-sensor technology such as global positioning systems (GPS) within the match to quantify the running load demands of the players. Our correlation analysis did however show that players with higher IPC-F, those who also tend to play in positions known to perform higher volumes of high-speed, sprint and high-acceleration distances in youth soccer [36], showed greater declines in IPC-F 30° at all time-points, and IPC-F 90° at all timepoints except post-match, suggesting that stronger players may require extended recovery before return to loading. Although, our study was limited by sample size and the lack of technology to monitor player-specific running loads, these observations represent interesting patterns which warrant further research.

While the greater HSR loads performed by the stronger players would be expected to play an important part in the negative correlation between IPC-F and IPC-F recovery following match-play observed, other factors such as greater damage to type 2 than type 1 fibres [39,40] may also contribute to the greater fatigue/slower recovery in players with greater IPC-F. Nonetheless, this finding does appear to run counter to the notion that higher strength is associated with greater resilience or robustness; i.e. the ability to cope with and recover from the demands of competition. To our knowledge, the association between hamstring/posterior chain strength and the recovery profile in that muscle group has not been previously examined. However, there is evidence that “physical qualities” including strength, in knee/hip extension may mediate the neuromuscular fatigue response to competition as measured in a triple extension activity—the countermovement jump (CMJ) [41,42]. In two studies by Johnston et al. [19,41] examining elite youth rugby league players in which changes in CMJ peak power were used to quantify fatigue, it was shown that higher levels of aerobic fitness/intermittent running performance (measured with the Yo-Yo Intermittent Recovery test) and muscle strength (defined by 3RM back squat) were associated with reduced fatigue/more rapid recovery of neuromuscular function. The authors noted that stronger players covered significantly higher (GPS assessed) total and high-speed distances than weaker players during competition, yet post-match, and +24 h and +48 h they displayed the same deficits in CMJ peak power as the weaker players, suggesting that higher strength conserves at least one aspect of neuromuscular function under fatigue allows more work to be done for the same level of fatigue [41]. Similarly, in elite senior soccer players, Owen et al. [42] found moderate to large negative correlations between lower limb power (in a fixed load squat) and serum creatine kinase, a marker of muscle damage temporally associated with the residual fatigue response to high-intensity competition. On face value, our observations conflict with the findings of these studies, as we found that higher relative IPC-F was associated with larger acute and residual IPC-F decrements. Potentially, maximum isometric strength in the posterior chain is not protective but higher maximum eccentric strength [2], already known to be protective against hamstring injury, might be. Furthermore, Johnston et al. [19], reported that higher dynamic strength was associated with lower CMJ peak power changes, while lower limb strength was not measured, limiting direct comparisons. Lastly, it is well established from repeated sprint/soccer-specific activity results in significantly greater acute fatigue in hamstring than quadriceps peak force (torque) [26,43]. This greater susceptibility to fatigue in the hamstrings muscle group may mean that other physical qualities, such as repeated sprint ability, aerobic capacity, or posterior chain local strength-endurance, are more important in determining fatigue-recovery profile in this muscle group. This may also suggest that in players with the highest HSR and match demands, overall aerobic and buffering capacity [43,44] and metabolic adaptations in the relevant muscle fibres may not only confer greater fatigue resistance during competition [45] but may also influence residual neuromuscular deficit/enhance recovery. 

One of the limitations of our study is the lack of day-to-day reliability of the IPC-F tests within the present cohort, therefore we cannot determine the smallest worthwhile change or other metrics for meaningful change. While good reliability of both IPC positions tested has been previously reported in professional soccer players [15]; dominant-leg 90° (CV = 4.3%, ICC = 0.95), non-dominant leg at 90° (CV = 5.4%, ICC = 0.95), and non-dominant leg at 30° (CV = 4.8%, ICC = 0.93) and for dominant leg at 30° (CV = 6.3%, ICC = 0.86), it is recommended that within-population reliability is assessed and signal-to-noise determined using internal data [46]. Furthermore, increased familiarisation and practice on a daily basis through the testing cycle could have contributed to the ‘positive adaptation’ relative to pre-match values at +72 h post-match rather than true change in neuromuscular function. Although the present study was conducted in an elite population, results may not be generalizable as testing was conducted with a small sample (n = 14) from one team and with only one match per player included in the analysis, although this is the case with majority of similar studies [11,15,17]. However, this was partly due to the strict inclusion criteria of match-recovery cycles with no training in the subsequent 72 h, which is also a strength of the study, in that it allowed us to characterize the response to the match alone—without the influence of varying superimposed training loads.

## 5. Conclusions

In elite youth soccer players, we observed significant reductions in post-match IPC-F following competition with significant deficits remaining at +24 h and recovery at +48 h, with evidence for positive adaptation at +72 h. There was substantial inter-individual variability in recovery kinetics, such that at +72 h there were players with IPC-F still below pre-match values, highlighting the importance of monitoring individual responses. IPC-F testing following match-play provides a simple and non-invasive mode to regularly monitor muscle force production at the hamstring complex and thereby quantify individual players recovery from matches and chronic trends in neuromuscular function. In a pro-active sports science/medical support model this provides data which may inform fast decision making around training load adjustment/recovery strategies in elite youth soccer players, assisting with player preparation and management, and potentially reducing HSI risk. Overall the two testing positions did not differ in their fatigue-recovery profile, but the 30° position appeared to have a higher sensitivity to acute fatigue. Nonetheless, as IPC-F testing is typically performed on a match-day plus two (i.e. +48 h) and at this timepoint differences between these positions were at their lowest, practical considerations may favour the use of the 90°. In environments like elite lower league youth soccer, where financial restrictions may restrict access to GPS technology, IPC-F performance evaluated using a low cost force platform such as in the present study, or a pressure cuff [11,24] or a fixed dynamometer [17], could provide information on the players neuromuscular response to match and training load in hamstring/posterior chain, and help to identify abnormalities in that response.

## Figures and Tables

**Table 1:** Mean (SD) for isometric posterior chain force (N/kg) at 30° and 90° and % change at 24 h, 48 h and 72 h versus pre-match (N = 14).

	Pre	Post	Δ vs Pre Match (%)	+24	Δ vs Pre Match (%)	+48	Δ vs Pre Match (%)	+72	Δ vs Pre Match (%)
30°	5.8 (1.5)	4.6 (1.2)	−20.7	5.1 (1.6)	−12.1	5.6 (1.5)	−3.4	6.8 (1.3)	+17.2
90°	6.5 (1.4)	5.8 (1.7)	−10.8	6.3 (1.2)	−3.1	6.8 (1.2)	+3.1	7.1 (1.3)	+9.2
